# Triphenyl­methyl benzoate

**DOI:** 10.1107/S160053680902889X

**Published:** 2009-07-29

**Authors:** Richard E. Sykora, Lane McDonald, Greg T. Spyridis

**Affiliations:** a Department of Chemistry, University of South Alabama, Mobile, AL 36688-0002, USA; b Department of Chemistry, Seattle Pacific University, Seattle, WA 98119-1997, USA

## Abstract

The title compound, C_26_H_20_O_2_, has long been known, but was not structurally characterized until now. It adopts the *Z* conformation and the atoms comprising the ester linkage are essentially coplanar (r.m.s. deviation of 0.0234 Å). The acyl C—O bond length of 1.470 (2) Å falls within the normal range seen for esters of tertiary alcohols and is below the value of 1.496 Å found in tri-*tert*-butyl­methyl 4-nitro­benzoate.

## Related literature

For related structures of sterically hindered esters, see: phenyl benzoate (Adams & Morsi, 1976 ▶), a 4-substituted *tert*-butyl benzoate (Fu *et al.*, 2008 ▶), tri-*tert*-butyl­methyl 4-nitro­benzoate (Cheng & Nyburg, 1978 ▶), and for esters of tertiary alcohols, see: Allen & Kirby (1984 ▶); Schweizer & Dunitz (1982 ▶). For the synthesis, see: Blicke (1923 ▶) and for ionic field studies in solutions of the title compound, see: Velazquez *et al.* (2006 ▶). For additional related references on the calculated absolute structure parameter and the conformations of esters, see: (Flack, 1983 ▶) and Pawar *et al.* (1998 ▶).
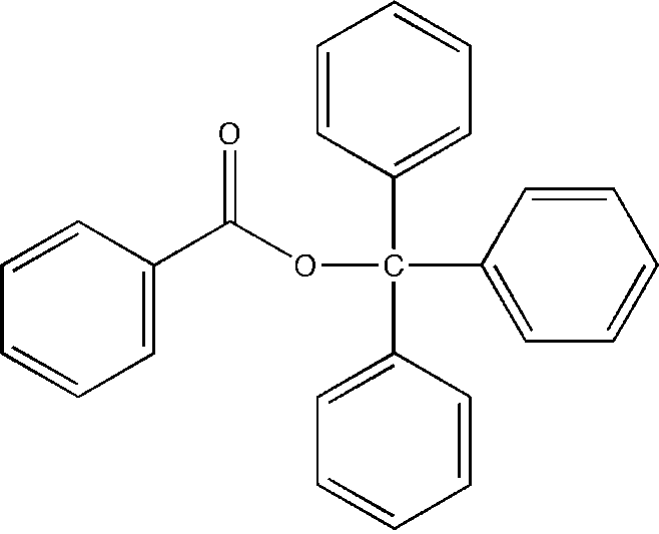


## Experimental

### 

#### Crystal data

C_26_H_20_O_2_
*M* *_r_* = 364.42
Orthorhombic, 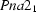

*a* = 8.9512 (4) Å
*b* = 14.9545 (5) Å
*c* = 14.4038 (6) Å
*V* = 1928.10 (13) Å^3^
*Z* = 4
Mo *K*α radiation
μ = 0.08 mm^−1^
*T* = 290 K
0.50 × 0.15 × 0.07 mm

#### Data collection

Oxford Diffraction Xcalibur diffractometer with an Eos CCD detector
Absorption correction: multi-scan (**SADABS**; Sheldrick, 1996 ▶) *T*_min_ = 0.959, *T*_max_ = 0.995
6656 measured reflections
2664 independent reflections
1879 reflections with *I* > 2σ(*I*)
*R*_int_ = 0.022

#### Refinement

*R*[*F* ^2^ > 2σ(*F* ^2^)] = 0.036
*wR*(*F* ^2^) = 0.070
*S* = 0.91
2664 reflections
254 parameters
1 restraint
H-atom parameters constrained
Δρ_max_ = 0.12 e Å^−3^
Δρ_min_ = −0.12 e Å^−3^

### 

Data collection: *CrysAlis Pro* (Oxford Diffraction, 2009 ▶); cell refinement: *CrysAlis Pro*; data reduction: *CrysAlis Pro*; program(s) used to solve structure: *SHELXS97* (Sheldrick, 2008 ▶); program(s) used to refine structure: *SHELXL97* (Sheldrick, 2008 ▶); molecular graphics: *SHELXTL* (Sheldrick, 2008 ▶); software used to prepare material for publication: *publCIF* (Westrip, 2009 ▶).

## Supplementary Material

Crystal structure: contains datablocks I, global. DOI: 10.1107/S160053680902889X/zs2001sup1.cif

Structure factors: contains datablocks I. DOI: 10.1107/S160053680902889X/zs2001Isup2.hkl

Additional supplementary materials:  crystallographic information; 3D view; checkCIF report

## Figures and Tables

**Fig. 1.**
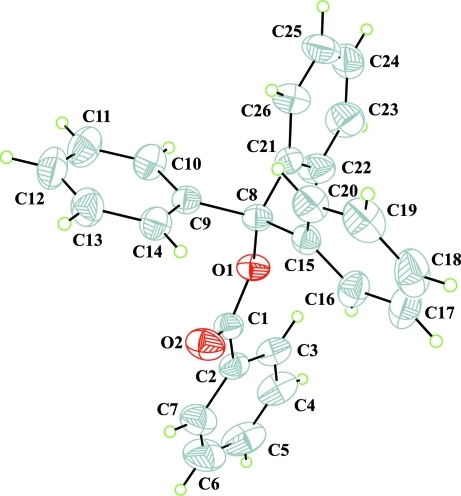
The molecular structure of (I), with the atom-numbering scheme. Displacement ellipsoids for non-hydrogen atoms are drawn at the 50% probability level.

## supplementary crystallographic information

### Comment

In light of our investigations into the use of the title compound as a probe of the ionic fields present in LiClO_4_-Et_2_O solutions, (Velazquez *et al.*, 2006), we prepared and crystallized triphenylmethyl benzoate, (I). The title compound adopts the *Z*-conformation (Pawar *et al.* 1998) with the C2—C1—O1—C8 dihedral angle being 3.5 (1)° and the phenyl ring exhibiting a slight twist of 17.6 (2)° with respect to the ester group. The atoms comprising the ester linkage, C2, C1, O2, O1 and C8, are essentially coplanar. The acyl C—O bond length of 1.470 (2) Å falls within the normal range as seen for the esters of tertiary alcohols (Allen & Kirby, 1984; Schweizer & Dunitz, 1982) and is well below the value of 1.496 Å in tri-*tert*-butylmethyl 4-nitrobenzoate (Cheng & Nyburg, 1978). The C1—O1—C8 bond angle is 120.50 (13)°, midway between the 118.3° observed in phenyl benzoate (Adams & Morsi, 1976) and the 122.9° seen in a 4-substituted *tert*-butyl benzoate (Fu *et al.* 2008), and is consistent with those noted for the esters of tertiary alcohols (Schweizer & Dunitz, 1982).

### Experimental

The title compound was synthesized by reacting trityl chloride with silver benzoate in dry benzene as outlined in the literature (Blicke, 1923). Crystals were grown by slow evaporation from benzene at 298 K. m.p. 442.5–444.15 K.

### Refinement

H-atoms were placed in calculated positions and allowed to ride during subsequent refinement, with *U*_iso_(H) = 1.2*U*_eq_(C) and C—H distances of 0.93 Å for all H atoms. The calculated absolute structure parameter (Flack, 1983) and e.s.d. was meaningless with a value of 0.1 (12). For this reason, the Friedel-pair reflections were merged before the final refinement.

### Crystal data

**Table:**

C_26_H_20_O_2_	*D*_x_ = 1.255 Mg m^−^^3^
*M**_r_* = 364.42	Melting point = 440.5–444.5 K
Orthorhombic, *P**n**a*2_1_	Mo *K*α radiation, λ = 0.71073 Å
Hall symbol: P 2c -2n	Cell parameters from 2913 reflections
*a* = 8.9512 (4) Å	θ = 3.1–30.4°
*b* = 14.9545 (5) Å	µ = 0.08 mm^−^^1^
*c* = 14.4038 (6) Å	*T* = 290 K
*V* = 1928.10 (13) Å^3^	Prism, colorless
*Z* = 4	0.50 × 0.15 × 0.07 mm
*F*(000) = 768	

### Data collection

**Table:**

Oxford Diffraction Xcalibur diffractometer with an Eos CCD detector	2664 independent reflections
Radiation source: fine-focus sealed tube	1879 reflections with *I* > 2σ(*I*)
graphite	*R*_int_ = 0.022
Detector resolution: 16.0514 pixels mm^-1^	θ_max_ = 30.4°, θ_min_ = 3.6°
ω scans	*h* = −11→6
Absorption correction: multi-scan (*SADABS*; Sheldrick, 1996)	*k* = −20→8
*T*_min_ = 0.959, *T*_max_ = 0.995	*l* = −14→20
6656 measured reflections	

### Refinement

**Table:**

Refinement on *F*^2^	Secondary atom site location: none
Least-squares matrix: full	Hydrogen site location: inferred from neighbouring sites
*R*[*F*^2^ > 2σ(*F*^2^)] = 0.036	H-atom parameters constrained
*wR*(*F*^2^) = 0.070	*w* = 1/[σ^2^(*F*_o_^2^) + (0.0394*P*)^2^] where *P* = (*F*_o_^2^ + 2*F*_c_^2^)/3
*S* = 0.91	(Δ/σ)_max_ < 0.001
2664 reflections	Δρ_max_ = 0.12 e Å^−^^3^
254 parameters	Δρ_min_ = −0.12 e Å^−^^3^
1 restraint	Extinction correction: *SHELXL97* (Sheldrick, 2008), Fc^*^=kFc[1+0.001xFc^2^λ^3^/sin(2θ)]^-1/4^
Primary atom site location: structure-invariant direct methods	Extinction coefficient: 0.0067 (13)

### Special details

**Table:**

Geometry. All e.s.d.'s (except the e.s.d. in the dihedral angle between two l.s. planes) are estimated using the full covariance matrix. The cell e.s.d.'s are taken into account individually in the estimation of e.s.d.'s in distances, angles and torsion angles; correlations between e.s.d.'s in cell parameters are only used when they are defined by crystal symmetry. An approximate (isotropic) treatment of cell e.s.d.'s is used for estimating e.s.d.'s involving l.s. planes.
Refinement. Refinement of *F*^2^ against ALL reflections. The weighted *R*-factor *wR* and goodness of fit *S* are based on *F*^2^, conventional *R*-factors *R* are based on *F*, with *F* set to zero for negative *F*^2^. The threshold expression of *F*^2^ > 2σ(*F*^2^) is used only for calculating *R*-factors(gt) *etc*. and is not relevant to the choice of reflections for refinement. *R*-factors based on *F*^2^ are statistically about twice as large as those based on *F*, and *R*- factors based on ALL data will be even larger.

### Fractional atomic coordinates and isotropic or equivalent isotropic displacement parameters (Å^2^)

**Table:**

	*x*	*y*	*z*	*U*_iso_*/*U*_eq_	
O1	0.09567 (13)	0.02387 (8)	0.19684 (8)	0.0377 (3)	
O2	0.27149 (15)	−0.04826 (9)	0.28069 (10)	0.0513 (3)	
C1	0.23123 (18)	−0.01384 (11)	0.20970 (13)	0.0364 (4)	
C2	0.3253 (2)	−0.00547 (11)	0.12520 (13)	0.0389 (4)	
C3	0.2950 (2)	0.05570 (13)	0.05580 (13)	0.0470 (5)	
H3	0.2109	0.0920	0.0602	0.056*	
C4	0.3891 (3)	0.06332 (16)	−0.02053 (15)	0.0611 (6)	
H4	0.3684	0.1045	−0.0671	0.073*	
C5	0.5127 (3)	0.0096 (2)	−0.02643 (17)	0.0715 (7)	
H5	0.5764	0.0146	−0.0772	0.086*	
C6	0.5431 (3)	−0.05147 (18)	0.0419 (2)	0.0729 (7)	
H6	0.6267	−0.0881	0.0367	0.087*	
C7	0.4512 (2)	−0.05927 (14)	0.11823 (16)	0.0564 (5)	
H7	0.4734	−0.1003	0.1648	0.068*	
C8	−0.01225 (19)	0.02868 (11)	0.27363 (12)	0.0340 (4)	
C9	−0.05440 (19)	−0.06734 (11)	0.30194 (12)	0.0352 (4)	
C10	−0.1514 (2)	−0.11564 (12)	0.24597 (14)	0.0486 (5)	
H10	−0.1927	−0.0888	0.1937	0.058*	
C11	−0.1874 (3)	−0.20348 (13)	0.26723 (17)	0.0612 (6)	
H11	−0.2518	−0.2353	0.2288	0.073*	
C12	−0.1287 (3)	−0.24365 (13)	0.34465 (17)	0.0589 (6)	
H12	−0.1538	−0.3024	0.3591	0.071*	
C13	−0.0327 (2)	−0.19667 (12)	0.40057 (16)	0.0517 (5)	
H13	0.0073	−0.2239	0.4530	0.062*	
C14	0.0052 (2)	−0.10936 (12)	0.37983 (14)	0.0424 (4)	
H14	0.0709	−0.0784	0.4182	0.051*	
C15	0.0481 (2)	0.08856 (11)	0.35106 (13)	0.0361 (4)	
C16	0.1604 (2)	0.15027 (12)	0.33333 (15)	0.0480 (5)	
H16	0.2058	0.1517	0.2753	0.058*	
C17	0.2052 (3)	0.20958 (14)	0.40161 (19)	0.0649 (7)	
H17	0.2819	0.2499	0.3894	0.078*	
C18	0.1379 (3)	0.20969 (14)	0.48721 (18)	0.0672 (7)	
H18	0.1681	0.2501	0.5326	0.081*	
C19	0.0260 (3)	0.14994 (15)	0.50529 (16)	0.0622 (6)	
H19	−0.0203	0.1500	0.5631	0.075*	
C20	−0.0189 (2)	0.08948 (13)	0.43834 (13)	0.0481 (5)	
H20	−0.0947	0.0489	0.4517	0.058*	
C21	−0.14691 (19)	0.07741 (10)	0.23019 (12)	0.0364 (4)	
C22	−0.1388 (2)	0.12181 (13)	0.14637 (15)	0.0545 (5)	
H22	−0.0505	0.1205	0.1124	0.065*	
C23	−0.2611 (3)	0.16830 (15)	0.11242 (19)	0.0677 (7)	
H23	−0.2539	0.1981	0.0559	0.081*	
C24	−0.3917 (2)	0.17089 (13)	0.1609 (2)	0.0619 (6)	
H24	−0.4733	0.2022	0.1377	0.074*	
C25	−0.4017 (2)	0.12700 (15)	0.24394 (18)	0.0601 (6)	
H25	−0.4905	0.1284	0.2774	0.072*	
C26	−0.2805 (2)	0.08062 (13)	0.27826 (16)	0.0512 (5)	
H26	−0.2889	0.0510	0.3348	0.061*	

### Atomic displacement parameters (Å^2^)

**Table:**

	*U*^11^	*U*^22^	*U*^33^	*U*^12^	*U*^13^	*U*^23^
O1	0.0315 (6)	0.0499 (6)	0.0316 (6)	0.0018 (5)	0.0016 (5)	0.0073 (6)
O2	0.0433 (7)	0.0647 (8)	0.0459 (8)	0.0086 (6)	−0.0012 (7)	0.0170 (7)
C1	0.0318 (9)	0.0397 (8)	0.0377 (10)	−0.0005 (7)	0.0008 (8)	0.0045 (8)
C2	0.0331 (9)	0.0452 (9)	0.0385 (10)	−0.0048 (8)	−0.0011 (8)	−0.0075 (9)
C3	0.0390 (11)	0.0613 (12)	0.0407 (11)	−0.0085 (9)	−0.0003 (8)	0.0032 (10)
C4	0.0582 (15)	0.0878 (15)	0.0373 (11)	−0.0193 (12)	0.0025 (11)	0.0017 (12)
C5	0.0568 (15)	0.109 (2)	0.0482 (13)	−0.0211 (14)	0.0167 (12)	−0.0187 (15)
C6	0.0523 (15)	0.0930 (18)	0.0732 (17)	0.0105 (13)	0.0152 (13)	−0.0237 (15)
C7	0.0464 (12)	0.0656 (12)	0.0572 (13)	0.0076 (10)	0.0028 (11)	−0.0081 (11)
C8	0.0313 (9)	0.0397 (9)	0.0310 (9)	0.0004 (7)	0.0020 (7)	0.0017 (8)
C9	0.0339 (9)	0.0363 (8)	0.0352 (10)	−0.0007 (7)	0.0051 (8)	−0.0005 (8)
C10	0.0560 (12)	0.0483 (10)	0.0414 (11)	−0.0051 (10)	−0.0024 (9)	−0.0026 (9)
C11	0.0712 (15)	0.0511 (12)	0.0611 (15)	−0.0175 (10)	0.0012 (12)	−0.0137 (12)
C12	0.0751 (15)	0.0384 (10)	0.0633 (13)	−0.0080 (10)	0.0131 (12)	0.0023 (11)
C13	0.0594 (13)	0.0429 (10)	0.0529 (13)	0.0007 (9)	0.0052 (10)	0.0099 (10)
C14	0.0442 (11)	0.0415 (9)	0.0416 (10)	−0.0015 (8)	0.0012 (8)	0.0026 (9)
C15	0.0369 (10)	0.0333 (8)	0.0380 (10)	0.0033 (7)	−0.0080 (8)	0.0034 (8)
C16	0.0545 (12)	0.0409 (9)	0.0488 (12)	−0.0062 (8)	−0.0097 (10)	0.0058 (9)
C17	0.0739 (16)	0.0410 (10)	0.0798 (18)	−0.0098 (10)	−0.0277 (14)	0.0023 (12)
C18	0.0845 (18)	0.0490 (11)	0.0682 (17)	0.0092 (12)	−0.0348 (14)	−0.0176 (12)
C19	0.0716 (16)	0.0685 (15)	0.0465 (12)	0.0149 (13)	−0.0105 (11)	−0.0109 (12)
C20	0.0520 (12)	0.0501 (11)	0.0421 (11)	0.0020 (9)	−0.0028 (9)	−0.0037 (10)
C21	0.0317 (9)	0.0365 (8)	0.0410 (11)	−0.0018 (7)	−0.0039 (8)	−0.0017 (8)
C22	0.0417 (11)	0.0644 (11)	0.0574 (13)	0.0009 (10)	−0.0059 (10)	0.0180 (12)
C23	0.0558 (14)	0.0701 (13)	0.0771 (17)	0.0055 (11)	−0.0182 (13)	0.0245 (13)
C24	0.0489 (13)	0.0501 (11)	0.0866 (18)	0.0090 (9)	−0.0288 (13)	−0.0054 (13)
C25	0.0361 (11)	0.0703 (13)	0.0739 (16)	0.0085 (10)	−0.0039 (11)	−0.0200 (13)
C26	0.0400 (10)	0.0628 (12)	0.0508 (12)	0.0055 (9)	0.0001 (10)	−0.0017 (11)

### Geometric parameters (Å, °)

**Table:**

O1—C1	1.351 (2)	C13—C14	1.382 (3)
O1—C8	1.470 (2)	C13—H13	0.9300
O2—C1	1.200 (2)	C14—H14	0.9300
C1—C2	1.486 (2)	C15—C16	1.389 (3)
C2—C3	1.382 (3)	C15—C20	1.393 (3)
C2—C7	1.388 (3)	C16—C17	1.384 (3)
C3—C4	1.390 (3)	C16—H16	0.9300
C3—H3	0.9300	C17—C18	1.373 (4)
C4—C5	1.370 (4)	C17—H17	0.9300
C4—H4	0.9300	C18—C19	1.367 (3)
C5—C6	1.369 (4)	C18—H18	0.9300
C5—H5	0.9300	C19—C20	1.382 (3)
C6—C7	1.378 (3)	C19—H19	0.9300
C6—H6	0.9300	C20—H20	0.9300
C7—H7	0.9300	C21—C22	1.380 (3)
C8—C15	1.529 (2)	C21—C26	1.383 (3)
C8—C9	1.540 (2)	C22—C23	1.386 (3)
C8—C21	1.541 (2)	C22—H22	0.9300
C9—C10	1.388 (3)	C23—C24	1.362 (3)
C9—C14	1.392 (3)	C23—H23	0.9300
C10—C11	1.387 (3)	C24—C25	1.368 (3)
C10—H10	0.9300	C24—H24	0.9300
C11—C12	1.372 (3)	C25—C26	1.379 (3)
C11—H11	0.9300	C25—H25	0.9300
C12—C13	1.371 (3)	C26—H26	0.9300
C12—H12	0.9300		
			
C1—O1—C8	120.50 (13)	C12—C13—H13	119.6
O2—C1—O1	124.44 (16)	C14—C13—H13	119.6
O2—C1—C2	124.32 (15)	C13—C14—C9	120.44 (19)
O1—C1—C2	111.22 (15)	C13—C14—H14	119.8
C3—C2—C7	119.40 (19)	C9—C14—H14	119.8
C3—C2—C1	122.47 (17)	C16—C15—C20	118.12 (17)
C7—C2—C1	118.09 (18)	C16—C15—C8	120.72 (17)
C2—C3—C4	120.5 (2)	C20—C15—C8	120.79 (16)
C2—C3—H3	119.8	C17—C16—C15	120.3 (2)
C4—C3—H3	119.8	C17—C16—H16	119.8
C5—C4—C3	119.4 (2)	C15—C16—H16	119.8
C5—C4—H4	120.3	C18—C17—C16	120.8 (2)
C3—C4—H4	120.3	C18—C17—H17	119.6
C6—C5—C4	120.4 (2)	C16—C17—H17	119.6
C6—C5—H5	119.8	C19—C18—C17	119.5 (2)
C4—C5—H5	119.8	C19—C18—H18	120.3
C5—C6—C7	120.8 (2)	C17—C18—H18	120.3
C5—C6—H6	119.6	C18—C19—C20	120.5 (2)
C7—C6—H6	119.6	C18—C19—H19	119.7
C6—C7—C2	119.5 (2)	C20—C19—H19	119.7
C6—C7—H7	120.2	C19—C20—C15	120.7 (2)
C2—C7—H7	120.2	C19—C20—H20	119.6
O1—C8—C15	110.20 (13)	C15—C20—H20	119.6
O1—C8—C9	108.33 (13)	C22—C21—C26	117.85 (18)
C15—C8—C9	116.08 (14)	C22—C21—C8	122.77 (16)
O1—C8—C21	103.39 (13)	C26—C21—C8	119.32 (16)
C15—C8—C21	107.19 (12)	C21—C22—C23	120.5 (2)
C9—C8—C21	110.90 (13)	C21—C22—H22	119.7
C10—C9—C14	118.24 (16)	C23—C22—H22	119.7
C10—C9—C8	119.00 (15)	C24—C23—C22	120.8 (2)
C14—C9—C8	122.71 (16)	C24—C23—H23	119.6
C11—C10—C9	120.7 (2)	C22—C23—H23	119.6
C11—C10—H10	119.7	C23—C24—C25	119.4 (2)
C9—C10—H10	119.7	C23—C24—H24	120.3
C12—C11—C10	120.3 (2)	C25—C24—H24	120.3
C12—C11—H11	119.8	C24—C25—C26	120.2 (2)
C10—C11—H11	119.8	C24—C25—H25	119.9
C13—C12—C11	119.56 (18)	C26—C25—H25	119.9
C13—C12—H12	120.2	C25—C26—C21	121.2 (2)
C11—C12—H12	120.2	C25—C26—H26	119.4
C12—C13—C14	120.7 (2)	C21—C26—H26	119.4
